# Canine Interferon-Inducible Transmembrane Protein Is a Host Restriction Factor That Potently Inhibits Replication of Emerging Canine Influenza Virus

**DOI:** 10.3389/fimmu.2021.710705

**Published:** 2021-10-14

**Authors:** Gang Lu, Jiajun Ou, Siqi Cai, Zhiying Lai, Lintao Zhong, Xin Yin, Shoujun Li

**Affiliations:** ^1^ College of Veterinary Medicine, South China Agricultural University, Guangzhou, China; ^2^ State Key Laboratory of Veterinary Biotechnology, Harbin Veterinary Research Institute, the Chinese Academy of Agricultural Sciences, Harbin, China; ^3^ Guangdong Provincial Key Laboratory of Prevention and Control for Severe Clinical Animal Diseases, Guangzhou, China; ^4^ Guangdong Technological Engineering Research Center for Pet, Guangzhou, China

**Keywords:** interferon-inducible transmembrane protein, canine, influenza virus, antiviral activity, IFITM

## Abstract

Canine influenza virus (CIV) is an emerging virus that is associated with major hidden hazards to the canine population and public health. Until now, how canine uses its innate immunity to restrict CIV replication is seldomly investigated. Recently, studies on interferon-inducible transmembrane (IFITM) of several major hosts of influenza virus (human, chicken, duck, pig) indicated it can potently restrict the viral replication. Here, the gene locus of five previously annotated canine IFITM (caIFITM) genes was determined on chromosome 18 using multiple bioinformatics strategies, provisionally designated as caIFITM1, caIFITM2a, caIFITM2b, caIFITM3, and caIFITM5. An analysis on protein sequences between caIFITM and its homologs indicated they shared the same conserved amino acids important for the antiviral activity. Expression profile analysis showed that caIFITM was constitutively expressed in tissues and MDCK cell line. After treatment with interferon or infection with influenza virus, the expression level of caIFITM increased with different degrees *in vitro*. An animal challenge study demonstrated CIV infection resulted in upregulation of caIFITM in beagles. caIFITMs had a similar subcellular localization to their human homologs. caIFITM1 was present at the cell surface and caIFITM3 was present perinuclearly and colocalized with LAMP1-containing compartments. Finally, we generated A549 cell lines stably expressing caIFITM and challenged them with influenza virus. The result demonstrated caIFITM1, caIFITM2a, caIFITM2b, and caIFITM3 had a potent antiviral activity against influenza virus. Our study will help better understand the evolutional pattern of IFITM and its role in the host’s defense against virus infection.

## Introduction

Types I and II interferons (IFNs) are produced when infected cells sense pathogen-associated molecular patterns and trigger cells to enter an antiviral state by inducing a variety of interferon-stimulated genes (ISGs). Although it is one of the first discovered ISGs, the antiviral activities of human interferon-inducible transmembrane [IFITM (huIFITM)] protein against pathogenic viruses had not been well characterized until 2009 (1). Using the siRNA screening strategy in osteosarcoma cells, it was found that knockdown of huIFITM proteins strongly promoted the replication of influenza virus *in vitro*. Further study found that huIFITM proteins have a broad antiviral activity against several other pathogenic viruses, including orthomyxoviruses, flaviviruses, filoviruses, coronaviruses, and alphaviruses (1–5). In addition, one study discovered that mice homozygous for IFITM3 deletion are more susceptible to influenza virus infection and exhibit more weight loss compared with the wild-type littermates (6). It was also reported that a synonymous rs12252 SNP in *huIFITM* gene alters a predicted splice acceptor site and is associated with the hospitalization in the 2009 H1N1 influenza pandemic (7).

To date, five homologs of IFITM (IFITM1, IFITM2, IFITM3, IFITM5, and IFITM10) have been found in humans on chromosome 11. IFITM5 is involved in bone mineralization and maturation (8). The function of IFITM10 remains unclear. IFITM1, IFITM2, and IFITM3 are associated with germ cell homing and maturation, tumor suppression, cell adhesion, and virus inhibition in innate immunity, among other functions (9). IFITMs are a family of small transmembrane proteins and contain five domains: an N-terminal domain (NTD) and a C-terminal domain (CTD), two intramembrane domains (IMD) of IM1 and IM2, and a conserved intracellular loop (CIL) (5). IFITMs share a highly conserved CD225 domain. A study on huIFITM3 shows that the CD225 domain is critical for the cellular localization (2). Three posttranslational modification patterns of palmitoylation, ubiquitination, and phosphorylation are associated with huIFITM3, and all contribute to its antiviral activity (10–12). In addition, the YXXΦ sorting motif at the N-terminus of huIFITM3 enables it to undergo endocytosis, thereby controlling both its endocytic trafficking and antiviral action (13).

The mode of the antiviral action of huIFITM3 is unique among restriction factors of innate immunity. huIFITM3 prevents virus cytosolic entry by blocking viral fusion subsequent to endocytosis and is considered the first line of antiviral defense of a cell (5). This protein plays an important role in antiviral actions of IFNs, accounting for 40% to 70% (1). In addition to huIFITMs, IFITMs from mouse, swine, chicken, and duck have also been investigated and are found to potently block influenza virus replication (4, 6, 14, 15). It is interesting that two swine IFITM1 proteins (IFITM1a, IFITM1b) are identified in swine cells, and all the studied five swine IFITM proteins (IFITM1a, IFITM1b, IFITM2, IFITM3, IFITM5) have antiviral activity (14). Chicken and duck IFITM proteins evolved as a single lineage and have more amino acid identity between each other compared with IFITM proteins from mammals (4, 15). Chicken IFITM2 and IFITM3 and duck IFITM3 show antiviral activity against several subtypes of influenza viruses (4).

Canine influenza virus (CIV) is responsible for canine influenza (CI) in the canine population. This virus emerged in the USA in 2004 and is currently prevalent worldwide (16, 17). CIV is an emerging threat to canine and public health. Until now, most studies on CIV have been associated with viral evolution and CI vaccine development. As a host of emerging influenza virus, how canine uses its innate immunity to restrict CIV replication is seldomly investigated. Although IFITMs from many different species have been well characterized as potent antiviral factors, whether canine encodes IFITM and whether canine IFITM (caIFITM) protein has antiviral activity against CIV remains unclear. In this study, we investigated the gene locus, expression pattern, and antiviral activity of caIFITMs.

## Materials and Methods

### Viruses and Cell Lines

Two H3N2 CIV strains A/canine/Guangdong/02/2014 (GD/2014) and A/canine/Guangdong/01/2018 (GD/2018) and one H5N1 CIV strain A/canine/Guangdong/01/2013 (GD/2013) were isolated from field sick dogs with respiratory diseases in Guangdong province in China. The virus was propagated in 9- to 11-day-old specific pathogen-free (SPF) embryonated chicken eggs and was stored at −80°C until further use.

Human-derived A549 and HEK293T and canine-derived MDCK cells were grown in Dulbecco’s modified Eagle’s medium (DMEM) (Life Technologies, Carlsbad, CA, USA) supplemented with 10% fetal bovine serum (BI) and 100 U penicillin and 100 U streptomycin (Life Technologies).

### *CaIFITM* Gene Locus

*CaIFITM* gene locus was flanked by telomeric-1,4-*N*-acetyl-galactosaminyl transferase 4 (B4GALNT4) and centromeric acid trehalase-like 1 (ATHL1) and was obtained through synteny analysis in the *Canis lupus familiaris* genome (CanFam3.1) in the GenBank database (https://www.ncbi.nlm.nih.gov/genbank/). The detailed *CaIFITM* gene position on canine chromosome was further determined using the web tool of NCBI Sequence Viewer 3.16.0 (http://www.ncbi.nlm.nih.gov/projects/sviewer/). BLASTN analysis was performed in the NCBI canine-expressed sequence tags (ESTs) or transcriptome shotgun assembly (TSA) sequence database to demonstrate the expression of *CaIFITM* gene.

### Plasmids

To obtain canine or human *IFITM* gene, total RNA was extracted from canine lung samples or HEK293T cells using RNAiso Plus (Takara, Dalian, China) and subjected to reverse transcription (RT). The *IFITM* genes were cloned from the synthesized cDNA by standard PCR using Phanta Super-Fidelity DNA Polymerase (Vazyme, Nanjing, China) and cloned into a the pCloneEZ-blunt-AMP plasmid vector using HC Cloning Kit (CloneSmarter, Houston, TX, USA) and then transformed into *E. coli* DH5α-competent cells (Weidi, Shanghai, China). After sequencing, *IFITM* genes were inserted into pEF4/myc-His plasmid (Life Technologies) with a Flag tag (DYKDDDDK) in the N-terminal by *Bam* HI*/Kpn* I and *EcoR* I digestion or inserted into the expressing GFP plasmid of pWPI (Addgene, Watertown, MA, USA) with a Flag tag in the N-terminal by *Bam* HI and *Spe* I digestion.

### IFITM-Expressing Cell Line Generation

Lentivirus vector stocks were made by a three-plasmid transfection into HEK293T cells. Briefly, HEK293T cells were seeded in six-well plates 1 day before transfection. For each well of HEK293T cells, 1 μg pMD.2G, 0.5 μg psPAX2, 0.5 μg empty pWPI-EGFP, or pWPI-EGFP constructs containing IFITM expressing frame were mixed with 200 μl Opti-MEM (Life Technologies). Another 6 μl Viafect transfection reagent (Promega, Madison, WI, USA) was added in the mixture and incubated for 20 min. The medium was removed and replaced with 2 ml DMEM plus 10% FBS. The mixture of plasmid and transfection reagent was then added in the cells. At 48 h after transfection, lentiviruses were collected and used to transduce A549 cells seeded in the six-well plates. Seventy-two hour after infection (hpi), A549 cells were trypsinized, seeded, and continuously cultivated in the 96-well plates to generate single-cell clones by the limiting dilution assay. The fluorescence expression in cell clones was observed by fluorescence microscopy.

### Real-Time PCR

Total RNA was extracted from canine cells or tissue samples using Minibest RNA extract Kit (Takara, Dalian). According to the manufacturer’s instructions, samples were treated with DNase I to remove genomic DNA. The obtained RNA was then reverse transcribed into cDNA using HiScript II Enzyme Mix (Vazyme, Nanjing), using oligo(dT)_23_ as a primer. IFITM, Mx1, canine IFN-α, and GAPDH mRNA expression level was estimated by quantitative reverse transcription PCR (RT-qPCR). Synthesized cDNAs were then subjected to RT-qPCR using a TB Green Premix Ex Taq II kit (Takara, Dalian) according to the manufacturer’s protocols. The RT-qPCR primer sets for each gene are listed in **Table 1**.

**Table 1:** Primers used for gene cloning and RT-qPCR analysis.

Gene	Primer sequences (5′→3′)	Product size (bp)	Purpose
caIFITM1	F^a^ : ACATCCGCAGTGACACG	200	RT-qPCR for detection of caIFITM1
	R^b^:CACGACCAAGGCCGAG		
caIFITM2a	F:AACGTTCCGGTGGAGA	217	RT-qPCR for detection of caIFITM2a
	R:TGGTCAGGAGGAGGC		
caIFITM2b	F:GTCCCGGAGGAGACG	210	RT-qPCR for detection of caIFITM2b
	R:GGATATGATGCCCAG		
caIFITM3	F:CCCCACCTACGAGATGC	93	RT-qPCR for detection of caIFITM3
	R:ATCACGGTGGTAATCG		
caIFITM5	F:CCAAAGCCAAGTGCTAC	159	RT-qPCR for detection of caIFITM5
	R:AGTCATAGTCCGAGTCCT		
Mx1	F:TGATATGCTGCACACGATAAC	181	RT-qPCR for detection of Mx1
	R:GATCTGCTCCATTTGGAAGTG		
CIV M	F:TGATCCTCTCGTTATTGCCGCAAG	159	RT-qPCR for detection of CIV
	R:CACTCTGCTGTTCCTGCCGATAC		
IFN-α	F:AACACGTCCTCTGCTCCTTG	153	RT-qPCRfor detection of IFN-α
	R:GTAGGTCCTCAGGGTGGAGT		
GAPDH	F:CATCACCATCTTCCAGGAGCG	146	RT-qPCR for detection of GAPDH
	R:AGATGATGACCCTTTTGGCT		
caIFITM1	F:ACACACAAACATCCCCA	479	Amplification of caIFITM1
	R:CGGAGCAGAGGGCTGGG		
caIFITM2a	F:CCTGTGCCTCCCCTCGAGA	480	Amplification of caIFITM2a
	R:GTCTGTGCACCCGGAGCAG		
caIFITM2b	F:TGGCCACCTGCACTCTT	441	Amplification of caIFITM2b
	R:GGGGCCCAGGGCGTCCT		
caIFITM3	F:GCACCTGCCACCATGAGCC	472	Amplification of caIFITM3
	R:AGGCCTCCGGCGCCGACTA		
caIFITM5	F:ATGGACACGGCGTACCCCCGC	527	Amplification of caIFITM5
	R:CTGGAGTCAGGGTTCCGGGCA		

^a^Forward; ^b^Reverse.

### Animal Experiment

A total of six 10-week-old beagles were used in the present study. Before performing animal experiments, nasal and rectal swabs and serum samples were collected from each animal and determined as negative for CIV. The animals were randomly classified into the control group and challenge group, with three animals in each group. In the challenge group, each animal was inoculated intranasally with 1 ml (10^6^ EID_50_) H3N2 CIV strain GD/2018. In the control group, each animal was inoculated with an equal volume of sterile PBS. At 1 day postinfection (dpi), all the animals were euthanized and their lung samples were collected. Total RNA was extracted from each sample and was further processed to determine the expression level of caIFITMs by RT-qPCR.

### Confocal Microscopy and Indirect Immunofluorescence

A549 cells were seeded on glass-bottomed coverslips (NEST) and were transiently transfected with IFITM expression constructs. At 24 h after transfection, the cells were fixed with 4% (vol/vol) paraformaldehyde-phosphate-buffered saline (PBS) and were then permeabilized and blocked with QuickBlock Blocking Buffer for Immunol Staining (Beyotime, Shanghai, China). The cell samples were incubated with anti-Flag monoclonal antibody (Sigma-Aldrich, St. Louis, MO, USA) to detect IFITM protein or antilysosome-associated membrane glycoprotein 1 (LAMP1) antibody (Abcam, Cambridge, UK) to detect endosomes, followed by incubation with Alexa Fluor 594-conjugated goat antirabbit polyclonal antibody (Abcam) or Alexa Fluor 488-conjugated goat antimouse polyclonal antibody (Abcam). The nuclear DNA was labeled with Fade-4 6-diamidino-2-phenylindole (DAPI) solution (Beyotime). The cells were finally visualized with a Leica DM-IRE2 confocal microscope.

A549 cells expressing Flag-tagged IFITMs grown in 24-well plates were detected by indirect immunofluorescence using the protocol for confocal microscopy. The cells were finally visualized with a fluorescence microscope.

### Virus Infection

A549 cells stably expressing IFITMs in 24-well plates were infected with H3N2 (GD/2014) and H5N1 (GD/2013) CIV strains at 0.1 and 1 MOI, cultured in DMEM. At 24 hpi, cell culture was harvested. Viral RNA was extracted and the expressing level of viral *M* gene was determined by RT-qPCR using specific primer (**Table 1**).

### Statistical Analysis

All experiments were performed in triplicate. Statistical significance was determined using the conventional Student’s *t*-test and calculated by GraphPad Prism software 6. A *p*-value of <0.05 was considered significant (^*^ *p* < 0.05; ^**^ *p* < 0.01).

### Ethics Statement

All procedures associated with the animal experiments were approved by the South China Agricultural University Experimental Animal Welfare Ethics Committee. All animal protocols were conducted in the negative pressure environment of the Experimental Animal Center of South China Agricultural University.

## Results

### Identification and Revision of Previously Annotated caIFITMs

The previously reported human, mouse, swine, chicken, and duck *IFITM* gene loci were all flanked by two genes: B4GALNT4 and ATHL1 (4, 15). We searched the two genes in the *Canis lupus familiaris* genome (CanFam3.1) in the GenBank database (https://www.ncbi.nlm.nih.gov/genbank/) and found both genes (GenBank accession no. and chromosome location: NC_006600.3: 25,520,155-25,531,377; NC_006600.3: 25,443,387-25,449,288) on chromosome 18 with a 8.8-kb sequence gap. Then, using genome synteny, we totally identified four previously annotated genes positioned between canine B4GALNT4 and ATHL1 genes by the web tool of NCBI Sequence Viewer 3.16.0 (http://www.ncbi.nlm.nih.gov/projects/sviewer/): caIFITM1 (Gene ID, LOC606890; GenBank accession no. XM_843371.4), caIFITM1 like (LOC483396; XM_540515.6), caIFITM1 like (LOC475935; XM_533144.6), and caIFITM1 (LOC483397; XM_540516.3). Considering the CD225 domain is highly conserved among vertebrate IFITM proteins, we also performed a sequence similarity search against amino acid sequence of huIFITM3 on chromosome 18 in *CanFam3.1* using BLASTP analysis. The results hit the same proteins containing the CD225 domain encoded by the four previously annotated genes identified in the synteny analysis.

**Figure 1:**
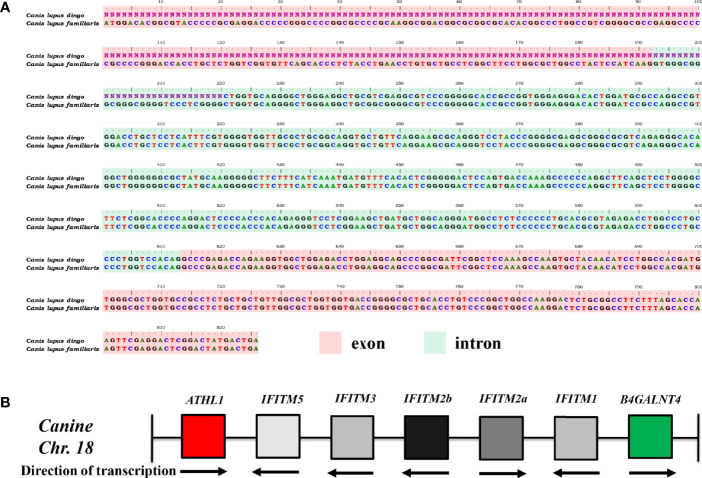
Identification of gene locus of caIFITMs in *CanFam3.1*. **(A)** Nucleotide alignment results of *caIFITM5* gene between *Canis lupus familiaris* and *Canis lupus dingo*. The exon and intron sequences were indicated by light red and light green, respectively. Nucleotide sequences that were not determined in *caIFITM5* gene in *Canis lupus dingo* were labeled “N.” **(B)** Organization of caIFITM locus on chromosome 18. *caIFITM* genes between ATHL1 and B4GALNT4 were annotated, and their direction of transcription were indicated.

It was noted that IFITM5 is not annotated in *CanFam3.1*. However, IFITM5 (Gene ID, 112663547; GenBank accession no. XM_025452624.2) was determined in dingo (*Canis lupus dingo*) genome (UNSW_AlpineDingo_1.0), locating between ATHL1 and other dingo IFITM genes. Dingo IFITM5 contains two exons and one intron (**Figure 1A**). Alignments results between dingo IFITM5 and DNA sequences (between ATHL1 and caIFITM1 [LOC606890]) of chromosome 18 in *CanFam3.1* demonstrated a same-exon sequence. However, gaps were found in the other exon sequence of IFITM5 in *CanFam3.1* (**Figure 1A**). This indicated canine genome possibly contains an *IFITM5* gene locus. Then, we performed a BLASTN analysis in the NCBI canine ESTs or TSA sequence database. The results indicated all the five putative *caIFITM* genes are expressed. Therefore, we have identified five putative *caIFITM* genes flanked by the genes *B4GALNT4* and *ATHL1* on chromosome 18. According to further analysis of caIFITM sequences, subcellular localization, and their antiviral function, we provisionally renamed previously annotated caIFITM locus in the NCBI database as follows: caIFITM1 (LOC483397) as caIFITM1, caIFITM1 like (LOC475935) as caIFITM2a, caIFITM1 like (LOC483396) as caIFITM2b, and caIFITM1 (LOC606890) as caIFITM3 (**Figure 1B**).

### Sequence Analysis of caIFITMs

**Figure 2:**
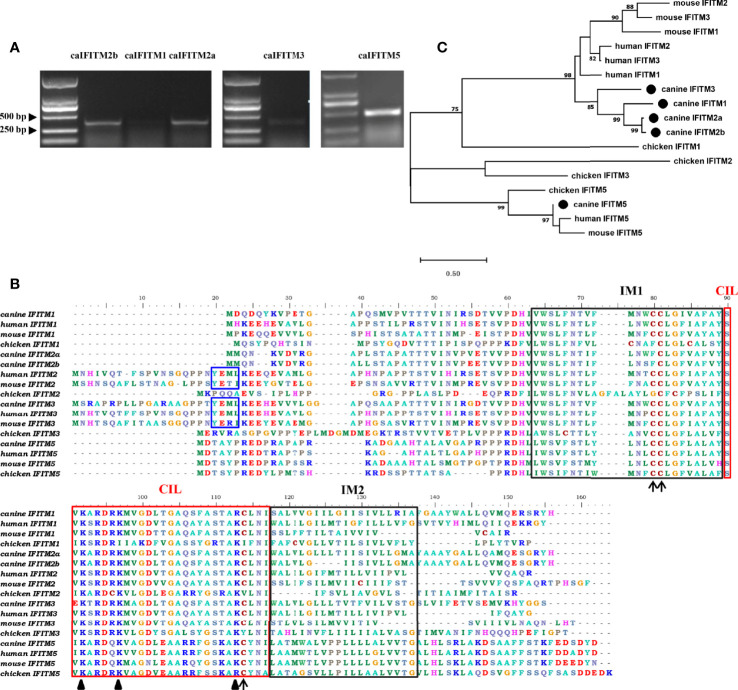
PCR amplification, sequence alignment, and phylogenetic analysis of caIFITMs. **(A)** Agarose gel electrophoresis result of caIFITMs. **(B)** Sequence alignment of caIFITMs with their homologs. The YXXΦ sorting motif, two TM regions, and CIL region were indicated by a box in blue, black, and red, respectively. The amino acids important for the antiviral activity of IFITMs were revealed by an arrow and a triangle, respectively. **(C)** Phylogenetic analysis of caIFITMs with their homologs. A maximum-likelihood tree was generated using MEGA 7.0 based on 1,000 bootstrap values. caIFITMs were indicated by a circle.

Primers targeting *caIFITM* genes were designed, and the corresponding fragments were amplified by RT-PCR from beagle lung samples. Gel electrophoresis results presented bands with expected sizes (**Figure 2A**). After sequencing and Blast analysis in the NCBI database, the results hit caIFITM1, caIFITM2a, caIFITM2b, caIFITM3, and caIFITM5, respectively. The raw sequence files of caIFITMs are provided in **Supplementary Material 1**. It was noted that caIFITM5 in beagles had a 100% nucleotide identity with *IFITM5* gene in dingo genome. caIFITM1, caIFITM2a, caIFITM2b, and caIFITM3 had a 51.0%–94.2% amino acid identity between one another and 29.0%–42.3% amino acid identity with caIFITM5. The highest amino acid identity (94.2%) was observed between caIFITM2a and caIFITM2b, with a total of eight amino acid differences between their coding sequences.

Amino acid sequences of the five caIFITM proteins were aligned with their chicken, human, and mouse homologs (**Figure 2B**). Like IFITM3 in human, mouse and chicken, caIFITM3 has a ~20 amino acids longer N terminus compared with other caIFITMs. caIFITM5 has a longer C terminus, which is found in all the analyzed vertebrate IFITM5 sequences. Three domains of IM1, IM2, and CIL are conserved in the analyzed IFITMs, especially among the mammalian homologs. The endocytosis motif YXXΦ is present in all the analyzed mammalian IFITM2 and IFITM3 sequences, which is also determined in caIFITM3 sequence. However, this motif was not found in caIFITM2a and caIFITM2b sequences. Several amino acids important for the antiviral activity of huIFITMs are conserved among caIFITMs, including palmitoylation site C81, ubiquitination sites K92 and K97, and C114 that are associated with antiviral activity against influenza virus. Palmitoylation site C81 is conserved in mammalian IFITM sequences, but an amino acid substitution at this site (C→F) was found in caIFITM2a and caIFITM2b sequences. Ubiquitination site K92 is also conserved in mammalian IFITM sequences. However, an amino acid substitution at this site (K→R) was determined in caIFITM1, caIFITM2a, caIFITM2b, and caIFITM3 sequences.

Analyzed by the phylogenetic tree (**Figure 2C**), it was found that caIFITM1, caIFITM2a, caIFITM2b, and caIFITM3 grouped most closely to the mammalian IFITMs, compared with the avian IFITMs. caIFITM5 was clustered together with other vertebrate IFITM5 in a clade, and the other five caIFITMs were located in another clade. In addition, caIFITM3 was clustered in a subclade by itself and caIFITM1b, caIFITM2a, and caIFITM2b were clustered with each other in another subclade.

### Expression Pattern of caIFITMs

**Figure 3:**
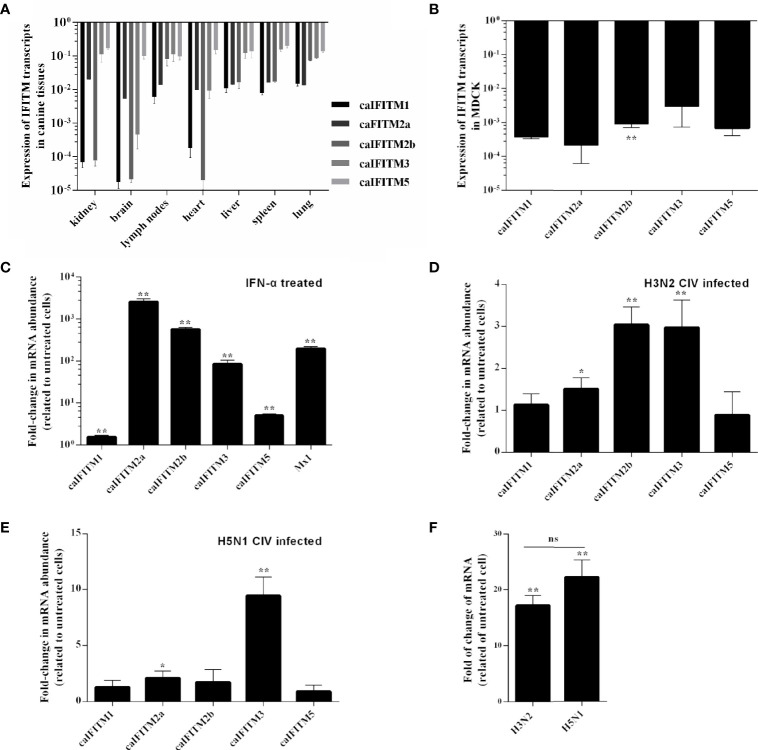
The expression pattern of caIFITMs. **(A)** The expression level of caIFITMs in tissue samples from beagles. Seven tissue samples were individually collected from three 10-week-old beagles. RNA was extracted from each sample and subjected to RT-qPCR. The expression level of caIFITMs in each tissue was compared with GAPDH. **(B)** The expression level of caIFITMs in MDCK. RNA was extracted from cultured MDCK cells and was used for RT-qPCR. The expression level of caIFITMs was compared with GAPDH. **(C)** The change of expression level of caIFITMs in MDCK after treated with IFN. MDCK cells were treated with recombinant human IFN-α (10,000 U). After 24 h, both treated and untreated MDCK cells were collected, and expression level of caIFITMs was determined by RT-qPCR. The expression level of caIFITMs of cells treated with IFN was compared with that in untreated cells. Mx1 was used as control. **(D, E)** The change of expression level of caIFITMs in MDCK after being infected with H3N2 CIV (GD/2014) and H5N1 CIV (GD/2013). MDCK cells were infected with H3N2 CIV (GD/2014) or H5N1 CIV (GD/2013) at 0.1 MOI. At 24 hpi, cells were collected and expression level of caIFITMs was determined by RT-qPCR. The expression level of caIFITMs of cells treated with CIV was compared with that in untreated cells. Mx1 was used as control. **(F)** The change of expression level of canine IFN-α in MDCK after being infected with H3N2 CIV (GD/2014) and H5N1 CIV (GD/2013). Cells collected in **(D, E)** were processed for estimating expression level of canine IFN-α by RT-qPCR. The expression level of canine IFN-α of cells treated with H3N2/H5N1 CIV was compared with that in untreated cells. All data represent the means and SD for experiments (each repeated in triplicate) and were analyzed by Student’s *t*-test. (ns, not significant; ^*^ *p* < 0.05; ^**^ *p* < 0.01).

To understand expression pattern of caIFITMs, we firstly determined tissue/cell-specific gene expression pattern of caIFITMs in seven different canine-derived tissues and MDCK cell line (**Figures 3A, B**). Tissue samples were collected from three beagles. Quantified by RT-qPCR, the expression level of caIFITM5 was high in each tissue. The lowest expression level of caIFITM was determined in caIFITM1 in brain. In addition, the expression level of caIFITMs was not constant between different tissues. RT-qPCR results showed most caIFITMs had an expression level of >10^−2^-fold of GAPDH, except mRNA expression of caIFITM1 and caIFITM2b in kidney, caIFITM1, caIFITM2a, caIFITM2b, and caIFITM3 in brain and heart, and caIFITM1 in lymph nodes and spleen. In MDCK, caIFITMs had an expression level of 10^−4^~10^−2^-fold of GAPDH. The expression levels of caIFITM2a, caIFITM3, and caIFITM5 in MDCK were equal to caIFITM1, and the expression level of caIFITM2b was significantly higher than caIFITM1 (*p* < 0.01).

Then, we estimated the expression pattern of caIFITMs *in vitro* when MDCK cells were treated with recombinant human interferon-α (IFN-α, 10,000 U) (**Figure 3C**). As a control, addition of interferon-α caused a near 400-fold increase in Mx1 expression in MDCK. The expression of all caIFITMs was upregulated (*p* < 0.01). However, it was observed that IFN-α induced the highest upregulation of about 2,500-fold of caIFITM2a expression and only caused about 1.5-fold of caIFITM1 expression.

Next, we investigated the expression of caIFITMs *in vitro* when MDCK cells were infected with H3N2 CIV (GD/2014, 0.1 MOI) and H5N1 CIV (GD/2013, 0.1 MOI) (**Figures 3D, E**). At 24 hpi, H3N2 CIV caused an increase in expression of caIFITM2a (1.5-fold, *p* < 0.05), caIFITM2b (3-fold, *p* < 0.01), and caIFITM3 (3-fold, *p* < 0.01). In addition, H5N1 CIV infection caused an increase in expression of caIFITM2a (2-fold, *p* < 0.05) and caIFITM3 (9-fold, *p* < 0.01). We then tested mRNA expression of canine IFN-α in MDCK cells infected with CIV (**Figure 3F**). It was found that both H3N2 CIV and H5N1 CIV could induce an increase of IFN-α mRNA expression level compared with untreated cells (*p* < 0.01). However, statistical analysis showed that there was no significant difference in IFN-α mRNA expression level between H3N2 CIV and H5N1 CIV infection groups (*p* > 0.05).

### Expression Pattern of caIFITMs in Animal Experiments

**Figure 4:**
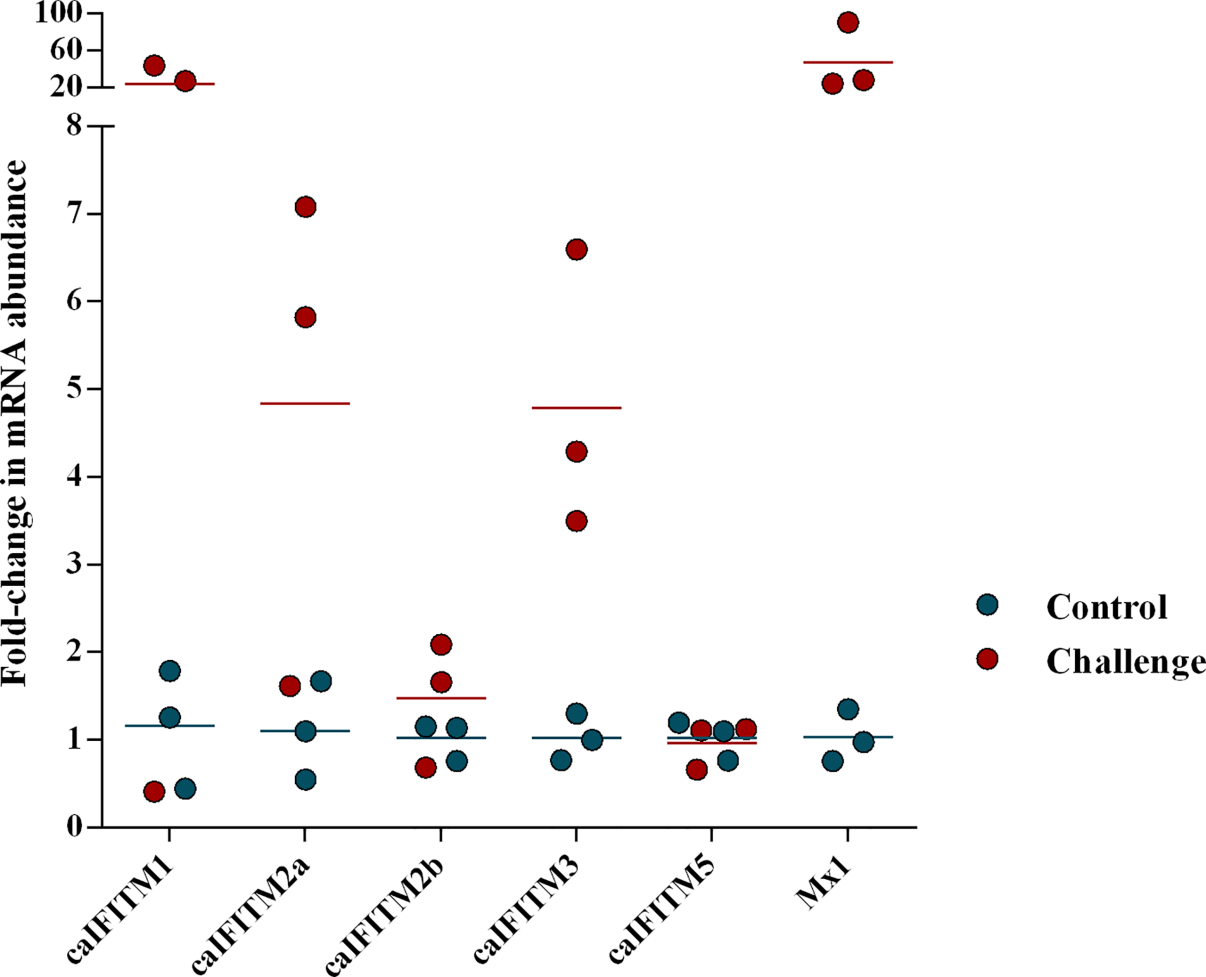
The change of expression level of caIFITMs in beagles after being challenged with H3N2 CIV. The experimental beagles (*n* = 3) were inoculated intranasally with 10^6^ EID_50_ of H3N2 CIV strain GD/2018. In the control group, beagles (*n* = 3) were treated with PBS in the same way. At 1 dpi, all the animals were euthanized. The expression level of caIFITMs in the lung samples were determined by RT-qPCR. All data were expressed as means ± SD.

Previous animal challenge studies demonstrated the expression level of certain host IFITM was upregulated after influenza virus infection. To determine change of caIFITM expression pattern in host response to infection with CIV, we challenged beagles with one H3N2 strain (GD/2018) (**Figure 4**). In the control group, beagles were treated with PBS. At 1 dpi, lung samples were collected from each animal and their caIFITM expression levels were examined using RT-qPCR. CIV infection caused 46-fold upregulation of Mx1 mRNA. caIFITM1 was highly up-regulated by 20-fold at 1 dpi. The expression of caIFITM2a, caIFITM2b, and dIFITM3 was modestly upregulated by 4-fold, 1.5-fold, and 5-fold respectively. No upregulation of caIFITM5 was observed in lungs.

### Subcellular Localization of caIFITMs

**Figure 5:**
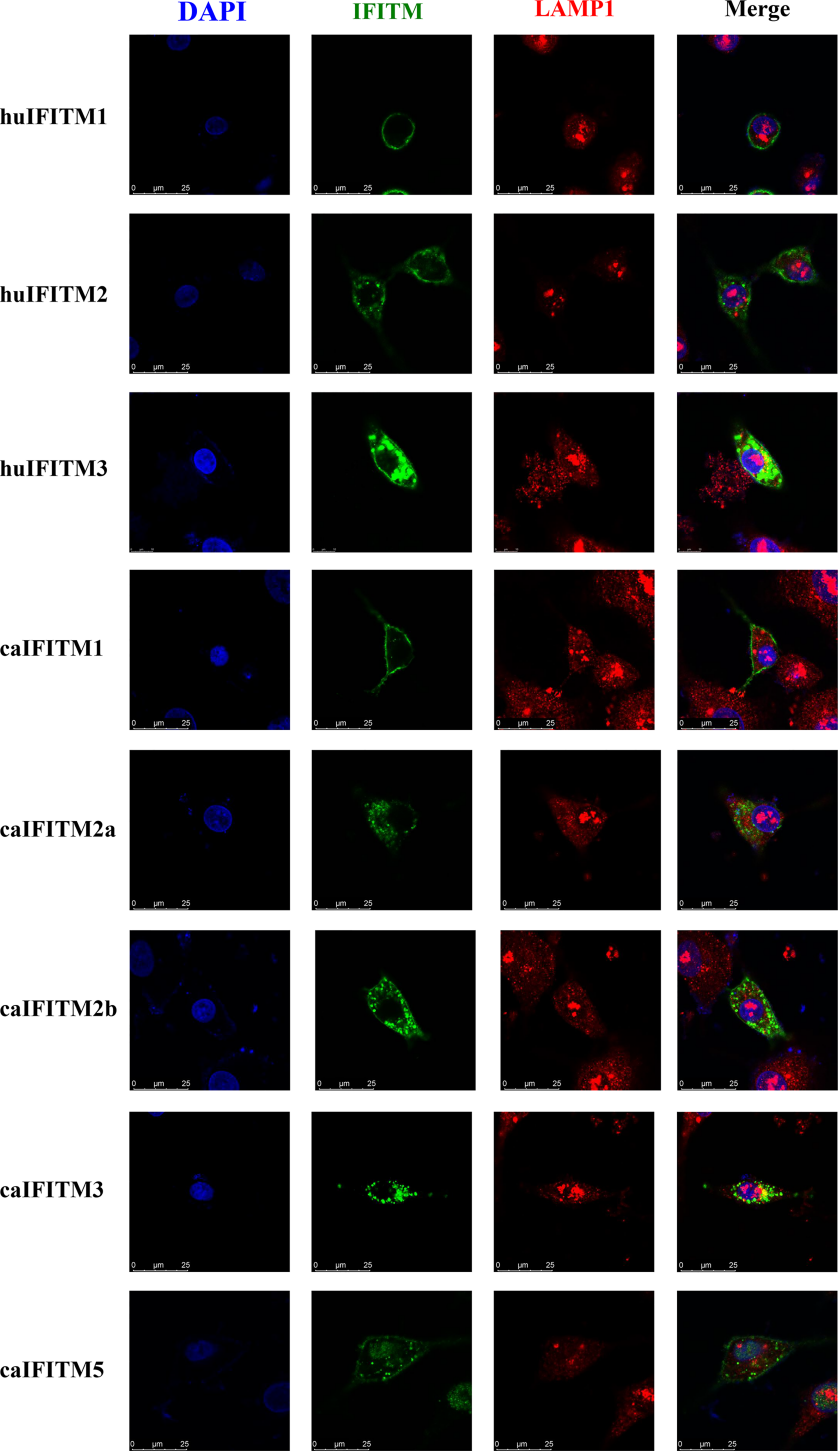
Subcellular localization of caIFITMs. A549 cells were transfected with Flag-tagged IFITM expression construct. After 24 h, the cells were stained with an anti-FLAG antibody (green) and an anti-LAMP1 antibody (red), and nucleus were stained with DAPI (blue). Finally, the cells were analyzed by a confocal microscopy.

To understand subcellular localization of caIFITMs, A549 cells were transiently transfected with three huIFITM and five caIFITM constructs with an N-terminal Flag tag (**Figure 5**). It was observed that huIFITM1 localized predominantly to cell surface, and huIFITM2 localized in cell surface and cytoplasm, wheras huIFITM3 was present perinuclearly. Confocal microscopy revealed that only huIFITM3 was colocalized with late endosomal marker LAMP1. caIFITMs localized to distinct cellular compartments. It was found caIFITM1 localized in cell surface, and caIFITM2a and caIFITM2b localized in cell surface and cytoplasm, whereas caIFITM3 was expressed perinuclearly. It was noted caIFITM5 localized in cell surface and cytoplasm and also in cell nucleus. Consistent with previous reports of mammalian and avian IFITM3 localization, caIFITM3 had colocalization with late endocytic compartments containing LAMP1, which was not observed with the other four caIFITMs.

### Antiviral Activity of caIFITMs

**Figure 6:**
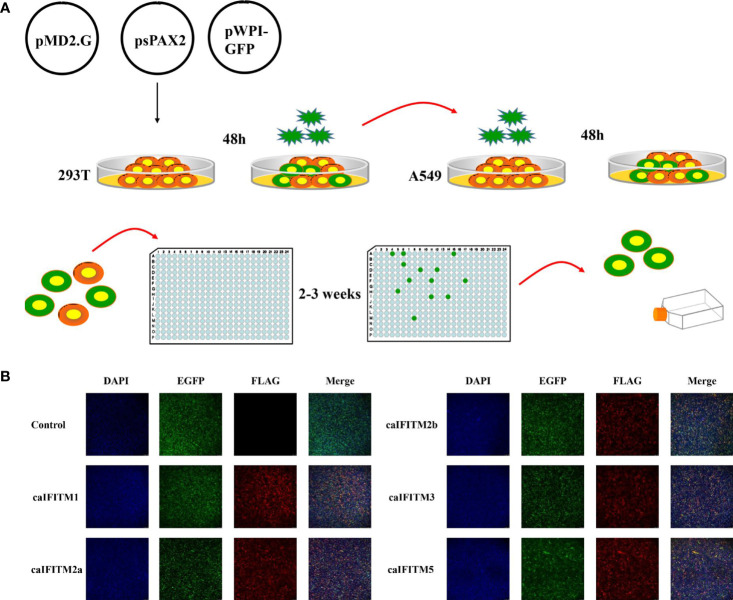
Establishment of A549 cell lines stably expressing caIFITMs. **(A)** Experimental process of generating and screening A549 cell lines stably expressing caIFITMs. HEK 293T cells were transfected with lentivirus packaging system. After 48 h, lentivirus in the cell culture supernatant was collected and was used for infecting A549 cells. After 48 h, A549 cells were trypsinized and seeded in 96-well plates by the limiting dilution assay and cultured for 2–3 weeks. Cell clones expressing fluorescence expression were selected by a fluorescence microscope. **(B)** Detection of caIFITM expression using the indirect immunofluorescence method. A549 cells stably expressing Flag-tagged caIFITMs were seeded on glass-bottomed coverslips. After 24 h, the cells were stained with an anti-FLAG antibody (red) and the nucleus was stained with DAPI (blue). Finally, the cells were analyzed by a fluorescence microscope.

A549 cells express low basal levels of endogenous IFITM and have been used to establish cell lines stably expressing IFITM to assess the impact of IFITM on influenza virus infection *in vitro* (1, 2). To estimate antiviral activity of caIFITMs, we tried to establish A549 cell lines continuously expressing Flag-tagged IFITMs using lentivirus infection method and the limiting dilution assay (**Figure 6A**). In addition, one control cell line generated by infection with lentivirus vector stocks produced by pMD.2G, psPAX2, and one empty pWPI-EGFP plasmid. Finally, we generated a total of six clonal A549 cell populations, including one control cell line and five caIFITM-expressing cell lines. Each of the clones was confirmed to express green fluorescence during the screening process, which was subcloned until >99% of cells were positive for expression. The expression of IFITMs in the established cell lines was confirmed using the indirect immunofluorescence method with anti-Flag monoclonal antibody (**Figure 6B**).

**Figure 7:**
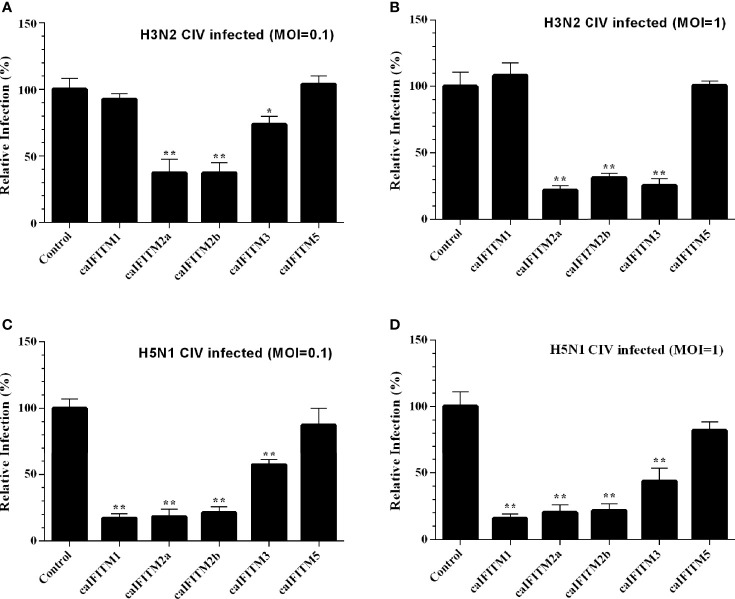
Antiviral activity of caIFITMs against CIV. A549 cells stably expressing caIFITMs were infected with H3N2 CIV (GD/2014) **(A, B)** and H5N1 CIV (GD/2013) **(C, D)** at 0.1 and 1 MOI. At 24 hpi, the cell culture supernatant was collected and used for RNA extraction. The expression level of CIV *M* gene was determined by RT-qPCR and was used for estimating the virus titer in cell culture supernatant. The virus titer of CIV harvested in A549 cells stably expressing caIFITMs was compared with the uninfected cell group. All data represent the means and SD for experiments (each repeated in triplicate) and were analyzed by Student’s *t*-test. (^*^ *p* < 0.05; ^**^ *p* < 0.01).

These six A549 cell lines were infected with H3N2 and H5N1 CIV at an MOI of 0.1 and 1 (**Figure 7**). Overexpression of caIFITM2a, caIFITM2b, and caIFITM3 resulted in 26%–78% reduction of H3N2 CIV viral replication. Whereas, overexpression of caIFITM1, caIFITM2a, caIFITM2b, and caIFITM3 reduced H5N1 CIV production by 43%–84%. caIFITM2a and caIFITM1 displayed the most potent antiviral activity against H3N2 and H5N1 CIV, respectively. However, caIFITM1 had no antiviral activity against H3N2 CIV in the present study.

## Discussion

Influenza virus is a major pathogen threatening the human and animal health. Its replication could be restricted by many cellular proteins, including several ISGs, such as myxovirus resistance A (MxA) (18), plasminogen activator inhibitor 1 (PAI-1) (19), bone marrow stromal cell antigen 2 (BST-2) (20), radical *S*-adenosyl methionine domain containing 2 (Viperin) (21), tripartite motif containing 22 (Trim22) (22), tripartite motif containing 25 (Trim25) (23), moloney leukemia virus 10 (Mov10) (24), and IFITM, among others. However, no systematic studies have been reported on the restriction function of canine ISGs on CIV replication. In this study, we identified the gene locus of caIFITMs and investigated their expression patterns and antiviral activities against CIV.

IFITM is classified in a large family of transmembrane proteins: *Dispanins* (25). IFITM homologs have been determined in vertebrate and invertebrate animals, and even prokaryotes (25–27). An evolutional analysis suggested that the origin of vertebrate IFITMs might be from a horizontal gene transfer from ancient bacteria species (25). The common characteristic of the IFITM homologs is that they all contain two-transmembrane helices structure and a highly conserved CD225 domain. In this study, we used huIFITM3 as the inquired protein, and successfully obtained five *caIFITM* genes flanked by *B4GALNT4* and *ATHL1* genes in canine genome database. A further analysis on their protein sequence also indicated they all contained TM1, TM2, and CD225 domain.

The vertebrate *IFITM* gene family is classified into three clades: clade I, II, and III. Clades II and III are composed of IFITM5 and IFITM10, respectively, while clade I contains other IFITMs (26). IFITMs in clade I are also named as immunity-related IFITM, because they could be induced by IFN and are associated with host immunity response (26). Our study showed that caIFITM1, caIFITM2a, caIFITM2b, and caIFITM3 were clustered together and caIFITM5 was clustered in another clade with other vertebrate *IFITM5* genes. As expected, the expression of caIFITM1, caIFITM2a, caIFITM2b, and caIFITM3 was induced by IFN and/or viral infection. Interestingly, the expression level of caIFITM5 also increased after IFN treatment, yet caIFITM5 could not inhibit replication of CIV. It has been reported that swine IFITM5 had inhibition activity against swine influenza virus and human influenza virus (14). The potential inhibition activity of caIFITMs against influenza virus derived from humans and other animals remains to be investigated.

The vertebrate IFITM underwent species-specific gene duplication during evolution (26). The genome of most vertebrates encodes three or more IFITMs (4, 14, 15). In a previous study, three canine IFITMs were predicted on chromosome 18 (26). Using multiple bioinformatics methods, we determined five caIFITM loci on chromosome 18 of the canine genome. IFITMs share high genetic similarity but could be distinguished from one another according to the characteristics of their protein sequence, subcellular localization, and antiviral activity: caIFITM5 was distinct from other caIFITMs in the protein sequence difference and phylogenetic clustering. IFITM3 possesses the endocytosis motif YXXΦ and generally has stronger colocalization with endocytic compartments containing LAMP1, which was also determined in caIFITM3. Notably, both caIFITM2a and caIFITM2b lack the endocytosis motif YXXΦ and are different from other vertebrate IFITM2 sequences. However, caIFITM1 was present at the cell surface and caIFITM2a and caIFITM2b localized in cell surface and cytoplasm, which could distinguish them from other caIFITMs.

It was noted that the fold changes of the mRNA abundance of caIFITM1 and caIFITM2a in some CIV-challenged beagles are similar to control (**Figure 4**). It may be caused by individual variations in beagles, such as distribution of influenza virus receptors in respiratory tract and basal level of innate-immunity-related genes, which could result in different virus replication level and mRNA abundance of caIFITMs in challenged individuals.

Until now, all the tested IFITMs in animal species have been determined having antiviral activity. In addition, it has been demonstrated that even mycobacterial IFITMs conferred resistance to influenza virus infection when transfected into 293T cells (27). Consistent with previous studies, caIFITMs were able to inhibit influenza virus infection. However, it was noted that caIFITM1 potently restricted replication of H5N1 CIV, but H5N1 CIV infection did not induce significant levels of caIFITM1 (**Figure 3E**). It is possible that H5N1 CIV inhibited the upregulation of caIFITM1 using certain antiviral mechanism to facilitate viral replication in host cells.

Our study determined the gene locus, expression pattern, and antiviral activity of caIFITM for the first time. A total of five caIFITM genes were acquired. Their expression level increased after induced by interferon or virus infection. Among them, caIFITM1, caIFITM2a, caIFITM2b, and caIFITM3 potently restricted the replication of CIV. In addition to influenza viruses, canines could infect a wide range of viral pathogens, such as canine parvovirus, canine distemper virus, canine coronavirus, and canine adenovirus. Further research is needed to understand the possible antiviral function and molecular mechanism of caIFITM against these viruses. Our study enriches the knowledge on caIFITM as one of the important canine ISGs.

## Data Availability Statement

The original contributions presented in the study are included in the article/**Supplementary Material**. Further inquiries can be directed to the corresponding authors.

## Ethics Statement

The animal study was reviewed and approved by South China Agricultural University Experimental Animal Welfare Ethics Committee.

## Author Contributions

Conceptualization: SL and XY. Methodology: GL. JO, and SC. Software: GL, JO, SC, and ZL. Validation: LZ, ZL, and JO. Formal analysis: GL. Investigation: GL. Resources: GL and XY. Writing (original draft preparation): GL. Writing (review and editing): XY and SL. Visualization: GL. Supervision: SL. Project administration: SL. Funding acquisition: GL. All authors contributed to the article and approved the submitted version.

## Funding

This work was supported by the National Natural Science Foundation of China (grant number: 31702271).

## Conflict of Interest

The authors declare that the research was conducted in the absence of any commercial or financial relationships that could be construed as a potential conflict of interest.

## Publisher’s Note

All claims expressed in this article are solely those of the authors and do not necessarily represent those of their affiliated organizations, or those of the publisher, the editors and the reviewers. Any product that may be evaluated in this article, or claim that may be made by its manufacturer, is not guaranteed or endorsed by the publisher.
